# Ratio of preoperative atrial natriuretic peptide to brain natriuretic peptide predicts the outcome of the maze procedure in mitral valve disease

**DOI:** 10.1186/1749-8090-8-32

**Published:** 2013-02-28

**Authors:** Masafumi Sato, Akihito Mikamo, Hiroshi Kurazumi, Ryo Suzuki, Masanori Murakami, Toshiro Kobayashi, Koich Yoshimura, Kimikazu Hamano

**Affiliations:** 1 Department of Surgery and Clinical Science, Division of Cardiac Surgery, Yamaguchi University Graduate School of Medicine, 1-1-1 Minami-Kogushi, Ube, Yamaguchi 755-8505, Japan; 2 Faculty of Nursing and Nutrition, Yamaguchi Prefectural University, 3-2-1 Sakurabatake, Yamaguchi, Yamaguchi 753-0021, Japan

**Keywords:** Atrial fibrillation, Cardiac surgery, Fibrosis, Natriuretic peptides

## Abstract

**Background:**

Although the maze procedure is an established surgical treatment for eliminating atrial fibrillation (AF), its efficacy in patients with mitral valve disease has remained unsatisfactory. A useful predictive marker for the outcome of the maze procedure is needed. The aim of this study was to investigate whether the preoperative ratio of atrial natriuretic peptide (ANP) to brain natriuretic peptide (BNP) reflects atrial fibrosis and can be used to predict the maze procedure outcome in patients with mitral valve disease.

**Methods:**

A total of 23 consecutive patients who underwent the radial approach to the maze procedure combined with mitral valve surgery were included in this study and were divided into a sinus rhythm (SR) group (n=16) and an AF group (n=7) based on postoperative cardiac rhythm. Plasma samples were obtained at rest before the operation and were analysed for ANP and BNP levels. Atrial tissue samples taken during surgery were used to quantify interstitial fibrosis.

**Results:**

The preoperative ANP-to-BNP ratio in the SR group was significantly higher than that in the AF group (0.74 +/− 0.29 vs. 0.42 +/− 0.28, respectively; p=0.025). Receiver operating characteristic (ROC) curve analysis was used to identify factors that predict outcomes after the maze procedure. The area under the ROC curve for the ANP-to-BNP ratio (0.81) was greater than for any other preoperative factors. Moreover, the preoperative ANP-to-BNP ratio demonstrated a negative correlation with left atrial fibrosis (r=−0.69; p=0.003).

**Conclusions:**

The preoperative ANP-to-BNP ratio can predict maze procedure outcome in patients with mitral valve disease, and it represents a potential biomarker for left atrial fibrosis.

## Background

Atrial fibrillation (AF) is the most common cardiac arrhythmia, especially for patients with mitral valve disease. The presence of postoperative AF is a significant risk factor for postoperative mortality, stroke, and other thromboembolism and anticoagulant-related haemorrhage [1]. Therefore, the restoration of sinus rhythm (SR) is critical for patients with AF and mitral valve disease who underwent mitral valve surgery. The maze procedure is an established surgical treatment for eliminating AF. The procedure reduces cardiovascular mortality and stroke and improves cardiac function [1,2]. Furthermore, its success rate for treating lone AF is higher than 90% [3]. However, only success rates between 60% and 90% have been reported with the maze procedure in patients with mitral valve disease [4,5]. Because the maze procedure has potential disadvantages, including prolonged operation and crossclamp times and the occasional need for implantation of a permanent pacemaker, performing this invasive procedure in patients who are less likely to receive a benefit should be avoided.

Atrial fibrosis plays an important role in developing and maintaining AF. Yoshihara et al. reported that the collagen volume in left atrial tissue was higher in AF than in SR [6]. However, previous risk factors for failure of the maze procedure, such as left atrial diameter (LAD), duration of AF and cardiothoracic ratio (CTR) [7,8], do not correlate well with atrial fibrosis [6,9]. Moreover, these risk factors do not fully predict the outcomes of the maze procedure. It has been reported that atrial natriuretic peptide (ANP) and brain natriuretic peptide (BNP) are related to atrial remodelling [6,10]. Furthermore, Mabuchi et al. reported that the ANP-to-BNP ratio was useful for predicting the recurrence of AF after direct current cardioversion in patients with mild chronic heart failure [11], which suggests that the ANP-to-BNP ratio may be useful for predicting the outcome of a maze procedure.

In the present study, we hypothesized that the preoperative ANP-to-BNP ratio more accurately predicts the outcome of a maze procedure in patients with mitral valve disease than the known risk predictors. Furthermore, we evaluated the ANP-to-BNP ratio as a biomarker for atrial interstitial fibrosis, which is a pathological process that has been associated with AF.

## Methods

### Patients

A total of 23 consecutive patients who had mitral valve disease (13 mitral regurgitation, 6 mitral stenosis and regurgitation, 4 mitral stenosis) and AF and underwent the radial approach to the maze procedure [12] at Yamaguchi University Hospital between April 2008 and December 2011 were examined. On the preoperative cardiac electrocardiogram, paroxysmal and chronic AF were documented in 1 and 22 patients, respectively. Left atrial enlargement was not used as an exclusion criterion. In addition, any patients with ischemic heart disease or chronic kidney disease were not included in this study. The cardiac rhythm after the maze procedure was assessed by electrocardiography (n=21) or 24 hour electrocardiographic monitoring (Holter) (n=2) 6 months after surgery and represented the maze procedure outcome. Actually, SR was restored in 16 of 23 patients (69.6%). To elucidate a predictive factor for maze procedure outcome, we divided the patients into SR (n=16) and AF groups (n=7) based on postoperative cardiac rhythm. Postoperatively, 2 patients with AF with slow ventricular response in the AF group and 1 patient with sick sinus symdrome in the SR group received permanent pacemakers. All protocols in the present study were approved by the Institutional Review Board of Yamaguchi University Hospital, and all patients gave written informed consent.

### Measurement of plasma ANP and BNP levels

Blood samples were taken at rest before the operation. Plasma ANP concentration was measured with a chemiluminescent enzyme immunoassay for α-human ANP (MI02 Shionogi ANP; Shionogi Co., Lts., Osaka, Japan) as previously described [13]. Plasma BNP concentration was measured with a chemiluminescent enzyme immunoassay for BNP (E Test TOSOH II (BNP); TOSOH Corporation, Tokyo, Japan) as previously described [14].

### Quantitative assessment of atrial fibrosis

We examined interstitial fibrosis samples from the left atrium, left atrial appendage, right atrium and right atrial appendage of 16 patients in this study. The quantitative collagen assay (No. 9046, Chondrex, Inc., Redmond, WA, USA) was performed as previously described [15]. In brief, atrial tissue samples were fixed in formalin and embedded in paraffin. Ten-micrometer tissue sections were stained with 0.1% Sirius red and 0.1% Fast green dissolved in water saturated with picric acid for 30 minutes. The epicardium, endocardium and microvessels in the sections were then carefully removed under a microscope. The colour eluted from the sections with a mixture of 0.1 N NaOH and methanol (1:1) was read in a spectrophotometer at 540 nm and 605 nm. The net amounts of total collagen and non-collagenous proteins were calculated using absorbances at 540 nm and 605 nm, respectively. The amount of total proteins was the sum of both absorbance values. Fibrosis was defined as follows: fibrosis (%) = (amount of collagen protein / amount of total proteins) × 100.

### Statistical analysis

Continuous data with was expressed as mean ± standard deviation. Categorical variables were reported as frequencies. Pre- and postoperative data were analysed using the unpaired *t*-test and Fisher’s exact test. The unpaired *t*-test was used for continuous variables with normal distribution. Fisher’s exact test was used for categorical variables. Receiver operating characteristic (ROC) curve analysis was used to identify factors that predict the outcomes of a maze procedure. Associations between continuous variables were assessed using correlation and linear regression techniques. P<0.05 was considered significant.

## Results

### Preoperative ANP-to-BNP ratio as a predictive factor for maze procedure outcome

To elucidate a predictive factor for maze procedure outcome, we compared preoperative variables between the SR (n=16) and AF groups (n=7). There were no significant differences in age, gender, CTR, left ventricular end-diastolic dimension (LVDd), ejection fraction (EF) and the preoperative ANP and BNP levels between two groups. In contrast, there was significant difference in LAD and the preoperative ANP-to-BNP ratio between the two groups (p=0.031, p=0.025, respectively). The preoperative ANP-to-BNP ratio was not identified as an independent factor by the multivariate analysis (p=0.059), but compared to the other factors, the ANP-to-BNP ratio demonstrated the most significant difference (Table 1).

**Table 1:** Preoperative clinical characteristics of patients

	**SR group (n=16**)	**AF group (n=7**)	**p value**
Age (years)	65.1+/−12.4	70.4+/−5.6	0.290^a^
Gender (male/female)	8/8	2/5	0.405^b^
CTR (%)	55.2+/−7.5	61.9+/−5.9	0.051^a^
LVDd (mm)	53.8+/−8.9	51.0+/−7.0	0.554^a^
LAD (mm)	51.5+/−10.6	63.5+/−13.5	0.031^a^
EF (%)	64.7+/−7.0	68.7+/−7.3	0.234^a^
Preoperative creatinine(mg/dl)	0.97+/−0.02	1.10+/−0.18	0.384^a^
Preoperative ANP (pg/ml)	120.1+/−67.3	74.8+/−45.4	0.141^a^
Preoperative BNP (pg/ml)	186.4+/−113.9	191.9+/−72.2	0.909^a^
Preoperative ANP/BNP ratio	0.74+/−0.29	0.42/-0.28	0.025^a^

^a^ Unpaired t test. ^b^Fisher’s exact test.

*AF*: atrial fibrillation; *ANP*: atrial natriuretic peptide; BNP: brain natriuretic peptide; *CTR*: cardiothoracic ratio; *EF*: ejection fraction; *LAD*: left atrial dimension; *LVDd*: left ventricular end-diastolic dimension.

**Figure 1:**
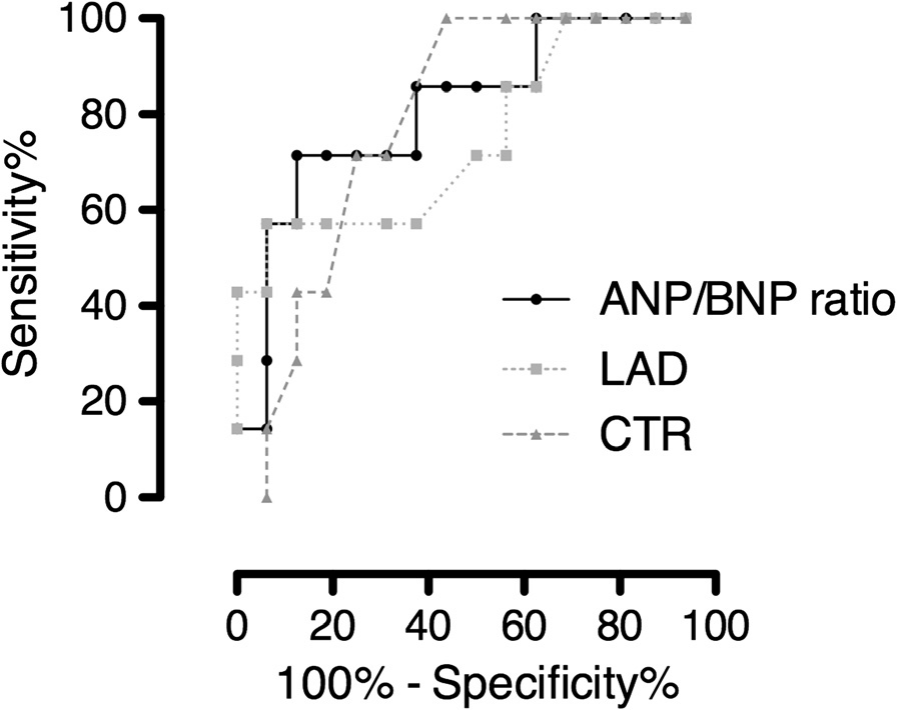
**ROC curve analysis in relation to the outcome after a maze operation.** ANP: atrial natriuretic peptide; BNP: brain natriuretic peptide; CTR: cardiothoracic ratio; LAD: left atrial dimension.

To demonstrate the usefulness of the preoperative ANP-to-BNP ratio as a predictive factor for maze procedure outcome, ROC analysis was performed on the preoperative ANP-to-BNP ratio as well as ANP level, BNP level, CTR and LAD. The area under the curve (AUC) for CTR and LAD was 0.79 and 0.75, respectively, indicating that CTR and LAD are also useful predictors of the outcome of a maze procedure, which is consistent with previous reports. Here, we found that the AUC for the preoperative ANP-to-BNP ratio was 0.81 or higher than AUC for ANP or BNP levels (Figure 1 and Table 2). Furthermore, the cutoff value for the preoperative ANP-to-BNP ratio to predict the restoration of sinus rhythm after a maze procedure was 0.41 (sensitivity 87.5%, specificity 71.4%). These findings indicate that the preoperative ANP-to-BNP ratio is the most useful for predicting the outcome of a maze procedure in patients with mitral valve disease.

**Table 2:** Area under the curve for ANP-to-BNP ratio, ANP, BNP, CTR and LAD

	**AUC**	**p value**	**95% confidence interval**
Preoperative ANP/BNP ratio	0.81	0.02	0.61 – 1.00
Preoperative ANP	0.73	0.08	0.50 – 0.96
Preoperative BNP	0.55	0.69	0.30 – 0.81
CTR	0.79	0.29	0.61 – 0.97
LAD	0.75	0.06	0.52 – 0.98

*ANP*: atrial natriuretic peptide; *AUC*: area under the curve; BNP: brain natriuretic peptide; *CTR*: cardiothoracic ratio; *LAD*: left atrial dimension.

### Correlation between the preoperative ANP-to-BNP ratio and atrial fibrosis

**Figure 2:**
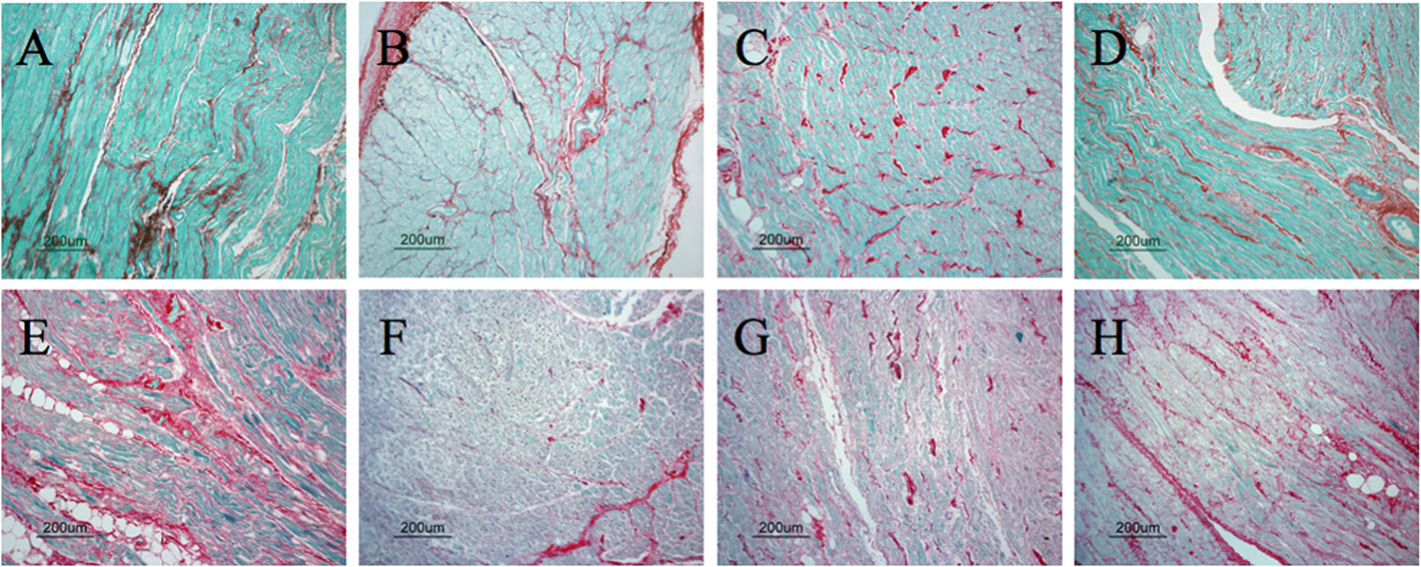
**Representative sections stained with 0.1% Sirius red and 0.1% Fast green in the left atrium (A, E), left atrial appendage (B, F), right atrium (C, G) and right atrial appendage (D, H) of patients with mild left atrial fibrosis (upper panel) and severe left atrial fibrosis (lower panel).** Scale bar=200 μm.

**Figure 3:**
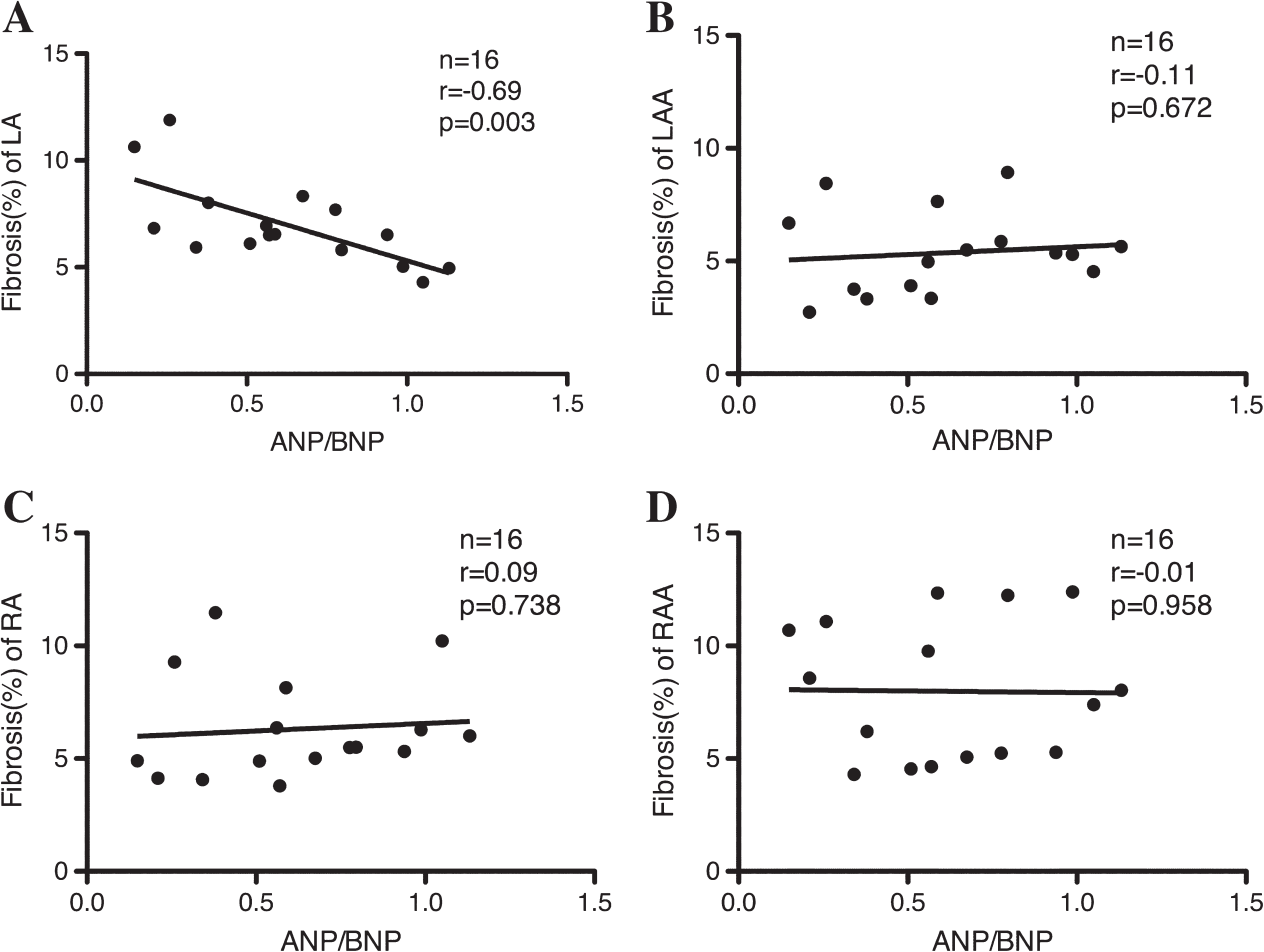
**Correlations between plasma ANP-to-BNP ratio and fibrosis (%) in the left atrium (A), left atrial appendage (B), right atrium (C) and right atrial appendage (D).** ANP: atrial natriuretic peptide; BNP: brain natriuretic peptide.

Next, we investigated the correlation between the preoperative ANP-to-BNP ratio and atrial fibrosis. Figure 2 shows representative sections stained with 0.1% Sirius red and 0.1% Fast green in the left atrium (A, E), left atrial appendage (B, F), right atrium (C, G) and right atrial appendage tissues (D, H) from patients with mild left atrial fibrosis (upper panel) and severe left atrial fibrosis (lower panel). In patients with mild and severe atrial fibrosis in the atrium, the degree of fibrosis was similar in the left atrial appendage, right atrium and right atrial appendage, but fibrosis (%) in the left atrium was 6.2% in one of SR and 11.3% in one of AF, respectively. Furthermore, in patients with mild and severe atrial fibrosis, the preoperative ANP-to-BNP ratios were 0.79 and 0.15, respectively. Interestingly, we found a negative correlation between the preoperative ANP-to-BNP ratio and fibrosis (%) in the left atrium (n=16, r=−0.69; p<0.003) (Figure 3A). In contrast, there was no correlation between the preoperative ANP-to-BNP ratio and fibrosis (%) in the left atrial appendage, right atrium or right atrial appendage (Figure 3B, C and D). In addition, neither ANP nor BNP levels showed a correlation with fibrosis (%) in any atrial tissues (data not shown).

**Figure 4:**
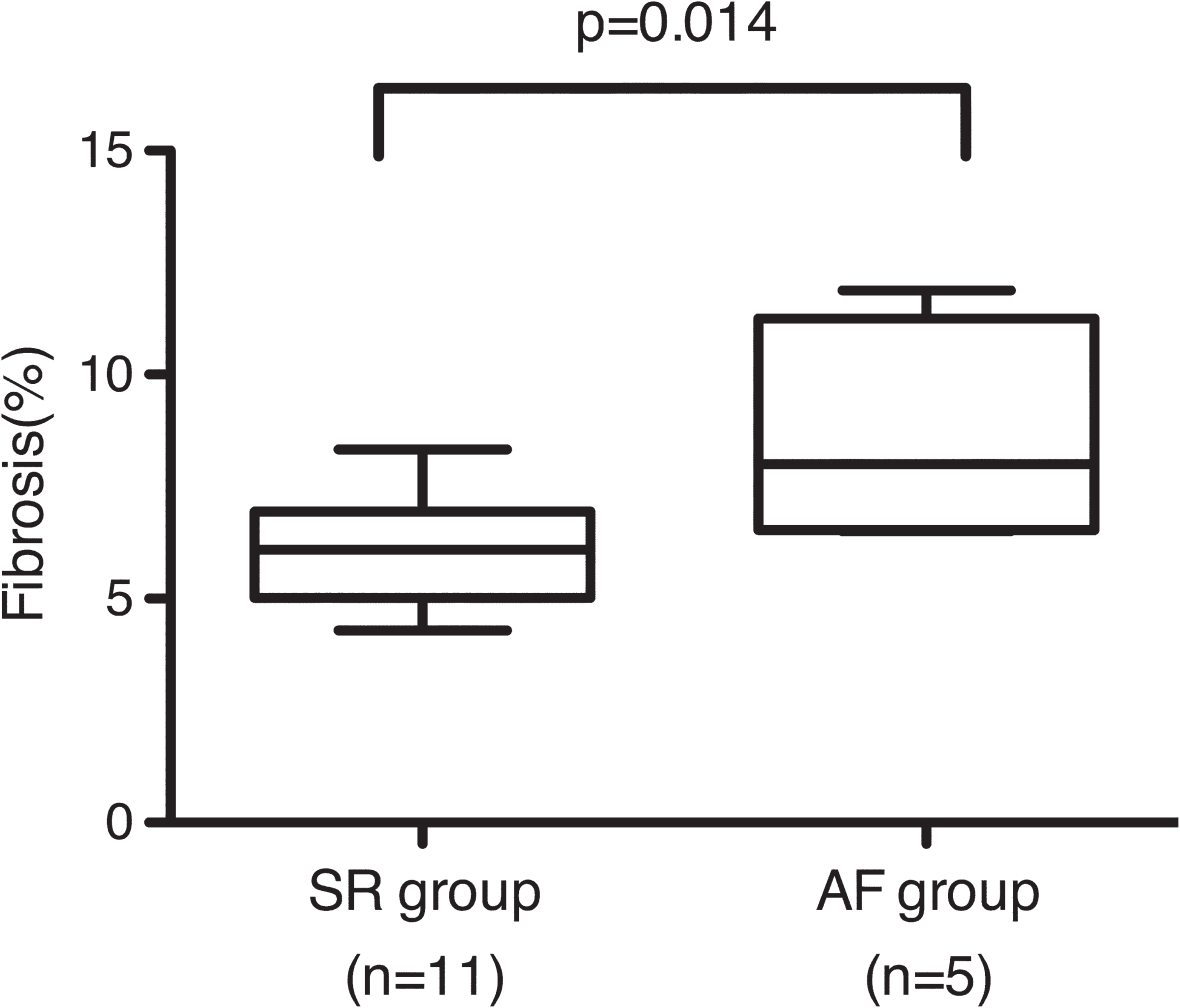
**Comparison between SR and AF groups for atrial fibrosis (%).** SR: sinus rhythm; AF: atrial fibrillation.

We also observed that left atrial fibrosis (%) in the AF group was significantly higher compared to that in the SR group (AF group, 8.7 ± 2.4%; SR group, 6.2 ± 1.2%; p=0.014, Figure 4). In contrast, mean fibrosis (%) in the left atrial appendage, right atrium and right atrial appendage in the AF group was slightly higher compared to that in the SR group (data not shown).

## Discussion

This is the first demonstration of a relationship between the preoperative ANP-to-BNP ratio and maze procedure outcome in patients with mitral valve disease. Atrial remodelling is important in maintaining AF. Intra-atrial conduction block, which leads to small and numerous reentrant circuits, is caused by increased atrial fibrosis [16,17]. The maze procedure was developed as a surgical treatment for AF. In the maze procedure, interruption lines are placed to block the reentrant circuits. Therefore, an unsuccessful maze procedure may be the result of smaller reentrant circuits. The maze procedure cures AF in most patients with lone AF [3]. In contrast, the success rates of the maze procedure for patients with AF and mitral valve disease, which induces the progression of left atrial remodelling, have been reported to be between 60% and 90% and have remained unsatisfactory [4,5]. Because the progression of mitral valve disease is associated with structural changes of the atrial wall, such as fibrosis in atrial tissues, the efficacy of the maze procedure is theoretically limited in the patients with severe mitral valve disease. In addition, the maze procedure is an invasive treatment and has several disadvantages, including prolonged operation time. This has raised concerns that this procedure may result in increased mortality in patient subsets less likely to benefit from the procedure. To avoid an unnecessary or excessive procedure, a useful predictive marker for maze procedure outcome is necessary, especially for patients with mitral valve disease. Hence, we sought to identify a preoperative factor that predicts maze procedure outcome. Large LAD and increased CTR were previously reported as risk factors for recurring AF after a maze procedure [7,8]. Yoshihara et al. reported that plasma ANP level also predicts maze procedure outcome [6]. In this study, CTR and plasma ANP levels were not predictors of maze procedure outcome. This may be due to the small sample size in our current study. Despite the small sample size, we have succeeded in identifying the preoperative ANP-to-BNP ratio as the most powerful predictor of maze procedure outcome. In addition, we showed that the preoperative ANP-to-BNP ratio was the best discriminator between patients with and without postoperative persistent AF (discriminator value=0.41). Using this cutoff value, the sensitivity and the specificity are 87.5% and 71.4% for predicting the restoration of sinus rhythm after a maze procedure. These results should lead to an increased success rate for the maze procedure. This strongly suggests that the preoperative ANP-to-BNP ratio is a promising tool for identifying the appropriate patients for the maze procedure.

We have also found that the preoperative ANP-to-BNP ratio is correlated with left atrial fibrosis. Although increased left atrial fibrosis is thought to be associated with the progression of mitral valve disease and failure of the maze procedure, the role of left atrial fibrosis in the development, recurrence and persistence of AF has not been fully elucidated. Saito and colleagues reported a correlation between the postoperative recurrence of AF and fibrosis in the left atrial appendage [18]. Kataoka et al. also reported that fibrosis in the left atrial appendage was significantly increased in patients in whom AF persisted after surgery [9]. Unfortunately, they did not assess left atrial interstitial fibrosis in their studies. In this study, we examined interstitial fibrosis in the left atrium, left atrial appendage, right atrium and right atrial appendage and found a considerable difference only in left atrial fibrosis between the SR and AF groups. Therefore, measuring left atrial fibrosis may be useful in determining the prognosis of patients with AF and mitral valve disease who underwent mitral valve surgery with a concomitant maze procedure. Moreover, the preoperative ANP-to-BNP ratio may also be a potential clinical biomarker for left atrial interstitial fibrosis.

Currently, the precise mechanism by which the preoperative ANP-to-BNP ratio is negatively correlated with left atrial fibrosis remains unknown. ANP is produced, stored and released by atrial cardiomyocytes in response to atrial stretch. It has been also reported that the decrease in plasma ANP is caused by the progression of atrial fibrosis [19]. This suggests that ANP by itself may be a potential biomarker for atrial fibrosis. Indeed, it has been reported that plasma ANP concentration inversely correlates with left atrial collagen volume fraction and is related to the outcome of a maze procedure [6]. However, in patients with mitral regurgitation or stenosis, there is a concern that ANP may be influenced not only by atrial fibrosis but also by an increased pressure or volume load in the atria [20]. By contrast, BNP is mainly produced and secreted by the ventricles in response to ventricular overload [20]. In patients with mitral regurgitation, plasma BNP increases with the severity of mitral regurgitation [21]. Mitral regurgitation causes volume overload with left atrial enlargement and left ventricular remodelling and induces BNP activation [10]. In patients with mitral stenosis, the degree of mitral stenosis has been shown to correlate with plasma BNP level, which is mainly produced by right ventricular myocytes due to right ventricular overload [22]. There is also positive correlation between plasma BNP level and LAD in patients with mitral stenosis [23]. As a result, ANP secretion in a patient with mitral valve disease is reduced according to the progression of the LA remodelling and enhanced overload, and the secretion of BNP is increased due to LA remodelling in patients with mitral regurgitation and stenosis. Therefore, the ratio of ANP to BNP may represent the degree of atrial fibrosis, that is necessary to counter the effect of pressure and volume load on the secretion of ANP. The ratio of ANP to BNP may be a better biomarker for atrial fibrosis than ANP by itself. In this study, we clearly demonstrated that the preoperative ANP-to-BNP ratio is much more useful in predicting the outcome of a maze procedure and assessing left atrial fibrosis than either plasma ANP or BNP alone.

This study has some limitations. Although we observed a significant correlation between the preoperative ANP-to-BNP ratio and maze procedure outcome, the sample size was quite small. Further studies with larger sample sizes are needed to establish the usefulness of the preoperative ANP-to-BNP ratio. We assessed the cardiac rhythm by electrocardiography in 21 patients and by 24-hour electrocardio-monitoring in the remaining 2 patients. Therefore, the success rate after the maze procedure may be overestimated. In a previous study, when the use of electrocardiography was compared with 24-hour electrocardio-monitoring, the detection rate of AF recurrence differed by 5.5%. However, the difference was not statistically significant [24]. In addition, even though we did not exclude such patients intentionally, no patients with ischemic heart disease or chronic kidney disease were enrolled in this study. BNP secretion was influenced not only by myocardial stretch but also by the ischemic insult [25]. It has also been reported that BNP is higher in patients with chronic kidney disease than in those without the disease [26]. These findings raise the possibility that the effectiveness of the preoperative ANP-to-BNP ratio may be decreased in patients with ischemic heart disease and/or chronic kidney disease. Therefore, the usefulness of preoperative ANP-to-BNP ratio in such patients should be examined in future studies.

## Conclusion

In conclusion, our findings demonstrate that the preoperative ANP-to-BNP ratio correlates negatively with left atrial fibrosis, predicts the outcome of a maze procedure in patients with mitral valve disease and represents a new biomarker that helps to decide when to perform a maze procedure.

## Competing interests

The authors declare that they have no competing interests.

## Authors’ contributions

MS developed study protocol, obtained data, analyzed data and wrote manuscript. AM developed the study protocol and provided critical revision of the manuscript. HK, RS, MM, TK, KY, and KH provided critical revision of the manuscript. All authors read and approved the final manuscript.
